# Checking for voice disorders without clinical intervention: The Greek and global VHI thresholds for voice disordered patients

**DOI:** 10.1038/s41598-019-45758-z

**Published:** 2019-06-27

**Authors:** Dionysios Tafiadis, Spyridon K. Chronopoulos, Meropi E. Helidoni, Evangelia I. Kosma, Louiza Voniati, Periklis Papadopoulos, Thomas Murry, Nafsika Ziavra, George A. Velegrakis

**Affiliations:** 1 Department of Speech & Language Therapy, University of Ioannina, Ioannina, GR45500 Greece; 2 Department of Health Sciences, Speech and Language Therapy, European University, P.O. Box: 22006-1516, Nicosia, Cyprus; 3 Physics Department, Electronics-Telecommunications and Applications Laboratory, University of Ioannina, Ioannina, GR-45110 Greece; 4 Department of Otolaryngology, University of Crete, Crete, Iraklion GR74100 Greece; 5 Psychologist, Private Practice, Napoleontos Zerva 2 & Pirsinella 1, Ioannina, GR45332 Greece; 6 Faculty of Medicine, School of Health Sciences, University of Thessaly, Biopolis, Larissa GR41500 Greece; 7 Physics Department, University of Ioannina, P.O. Box 1186, GR-45110 Ioannina, Greece; 8 Department of Otolaryngology-Head and Neck Surgery at Loma Linda University, CA 92354 California, USA; 9 Voice and Swallowing Centre, Loma Linda University, CA 92354 California, USA

**Keywords:** Oral diseases, Prognosis, Outcomes research

## Abstract

Voice disorders often remain undiagnosed. Many self-perceived questionnaires exist for various medical conditions. Here, we used the Greek Voice Handicap Index (VHI) to address the aforementioned problem. Everyone can fill in the VHI questionnaire and rate their symptoms easily. The innovative feature of this research is the global cut-off score calculated for the VHI. Therefore, the VHI is now capable of helping clinicians establish a more customizable treatment plan with the cut-off point identifying patients without normal phonation. For the purpose of finding the global cut-off point, a group of 180 participants was recruited in Greece (90 non-dysphonic participants and 90 with different types of dysphonia). The voice disordered group had higher VHI scores than those of the control group. In contrast to previous studies, we provided and validated for the first time the cut-off points for all VHI domains and, finally, a global cut-off point through ROC and precision-recall analysis in a voice disordered population. In practice, a score higher than the well-estimated global score indicates (without intervention) a possible voice disorder. Nevertheless, if the score is near the threshold, then the patient should definitely follow preventive measures.

## Introduction

The subjective voice evaluation using self-perceived questionnaires has been proven to effectively record the patients’ experience of their voice disorder, which has an impact on their quality of life (QOL)^1–3^. The European Laryngeal Society (ELS)^4,5^ included this type of evaluation in a basic voice assessment protocol. This protocol included endoscopy^6–8^ and acoustic, auditory-perceptual and aerodynamic evaluations^9–18^ after voice therapy or phonosurgical treatments^19,20^.

In particular, for the subjective assessment of the voice, one of the most prominent questionnaires is the Voice Handicap Index (VHI)^1^. The VHI is a self-questionnaire that consists of 30 items divided into three domains: functional (VHI-F), physical (VHI-P) and emotional (VHI-E)^1^. It has a total score (VHI-T) ranging from 0 to 120, divided equally over the three aforementioned domains.

After its release, the VHI was administered to populations with different types of voice disorders^21–33^, to patients before and after treatment (surgical and phonotherapy)^34–36^ and to populations with a risk of developing voice disorders^16–18^. VHI has been standardized in many languages^21–33^ and has been evaluated in comparison to other laboratory measurements^17,36^. Recently, VHI was employed on a mobile platform as a monitoring tool for vocal hygiene, proving its usefulness even via electronic means^37–39^. Although VHI was initially developed to obtain the opinions of patients regarding the psychosocial effects of their voice disorders on their daily lives^1^, recent studies have also reported its discriminatory ability^40–46^. The latter is a very important finding, as it demonstrates that this questionnaire is a very important tool in the hands of specialists and every person who wishes to preliminarily check his/her vocal-voice status for potential abnormalities before the use of interventional actions (e.g., endoscopy).

The aforementioned discriminatory capability has also been reported by studies that included the cut-off points for diverse populations^40–46^. Those cut-off points were computed with the use of the Receiver Operating Characteristic (ROC) analysis. The ROC analysis was developed by physicists during the Second World War and was used as an accurate discriminatory method for radar signal analysis. Similarly, this well-established method is currently used by health sciences to distinguish between healthy populations and populations with different pathologies. Specifically, the ROC curve is used to determine the appropriate threshold. Thus, the first purpose of our study was to determine the cut-off point for the VHI and its three domains for a voice disordered population from Greece (limitations apply, please see section 4.4). The second purpose of this study was to determine the correlations between various populations, while the third purpose was to estimate a global cut-off point (for voice disordered patients). The latter could be a true innovation in global voice screening procedures.

## Results

The sample consisted of 180 participants (90 controls and 90 voice disordered People - VDP). Non-dysphonic participants exhibited a VHI-T mean of 14.73 (SD = 2.27), a VHI-F mean of 4.56 (SD = 1.57), a VHI-P mean of 5.28 (SD = 1.78) and a VHI-E mean of 4.90 (SD = 1.63). The VHI-T mean score for the VDP group was 37.82 (SD = 20.89), while for the VHI-F group it was 10.72 (SD = 7.62), for the VHI-P group it was 16.98 (SD = 8.08), and for the VHI-E group it was 10.12 (SD = 8.10). The aforementioned are presented in detail in Table 1.

**Table 1:** Mean Scores for Control and VDP Subgroups for the VHI-T, VHI-F, VHI-P and VHI-E Domains.

	VHI-T	VHI-F	VHI-P	VHI-E
	Mean (SD) [Min–Max]	Mean (SD) [Min–Max]	Mean (SD) [Min–Max]	Mean (SD) [Min–Max]
Control group (N = 90)	14.73 (2.27) [11–21]	4.56 (1.57) [2–8]	5.28 (1.78) [1–9]	4.90 (1.63) [0–9]
LML (N = 40)	32.80 (14.09) [10–72]	8.10 (5.83) [0–20]	16.78 (6.14) [4–32]	7.93 (6.52) [0–28]
LID (N = 22)	28.27 (16.63) [4–57]	7.68 (5.21) [0–19]	12.45 (7.48) [2–24]	8.14 (7.03) [0–27]
NVD (N = 24)	55.67 (21.06) [7–97]	18.04 (7.13) [1–30]	22.04 (7.56) [6–35]	15.58 (8.69) [0–36]
HVD (N = 4)	33.50 (38.21) [0–70]	9.75 (10.72) [0–20]	13.50 (15.78) [0–30]	10.25 (11.84) [0–21]

Abbreviations: SD: Standard Deviation; VHI-T: Voice Handicap Index Total; VHI-F: Voice Handicap Index Functional; VHI-P: Voice Handicap Index Physical; VHI-E: Voice Handicap Index Emotional; LML: Laryngeal Mass Lesions; LID: Laryngeal Inflammatory Disorders; NVD: Neurogenic Voice Disorders; HVD: Hyper-functional Voice Disorders.

VHI can detect dysphonic patients or people with potential voice problems with the use of statistical, ROC, and precision-recall analysis. More details are given below.

To compare non-dysphonic participants with VDP, we conducted a Mann-Whitney test for the VHI-T and its three domains. In particular, statistically significant differences were observed between the two groups for VHI-T [U = 1204.500, P < 0.001], VHI-F [U = 2144.000, P < 0.001], VHI-P [U = 910.000, P < 0.001], and VHI-E [U = 2684.000, P < 0.001] (Table 2).

**Table 2:** Comparisons of Medians between Controls and VDP for the VHI Total Score and VHI Domains.

	Controls (N = 90)	VDP (N = 90)		
	Median (IQR)	Median (IQR)	Mann-Whitney U	P level
Total	15.00 (13.00–17.00)	35.50 (21.00–51.50)	1204.500	<0.001*
Functional	5.00 (3.00–6.00)	10.00 (4.75–16.00)	2144.000	<0.001*
Physical	5.50 (4.00–6.25)	18.00 (11.75–23.00)	910.000	<0.001*
Emotional	5.00 (4.00–5.00)	8.00 (4.00–16.00)	2684.000	<0.001*

*p level at P < 0.05; Abbreviations: IQR, Interquartile Range; VHI, Voice Handicap Index. VDP: Voice Disordered Population.

A Kruskal-Wallis test was conducted to compare the subgroup median VHI-T scores and the scores on the different domains. In particular, significant differences were observed between the non-dysphonic and dysphonic groups for the VHI-T score, with mean scores of 58.88 for non-dysphonic participants, 119.14 for LML patients, 102.30 for LID patients, 151.21 for NVD patients and 86.00 for HVD patients [H (4) = 79.272, P < 0.001] (Table 3).

**Table 3:** Comparisons of Medians between Study Subgroups for the VHI Total Score and VHI Domains.

	VHI-T	VHI-F	VHI-P	VHI-E
	Median (IQR)	Median (IQR)	Median (IQR)	Median (IQR)
**Controls (N** = **90)**	**15**.**00** (13.00–17.00)	**5**.**00** (3.00–6.00)	**5**.**50** (4.00–6.25)	**5**.**00** (4.00–5.00)
**LML (N** = **40)**	**32**.**00** (23.00–44.50)	**6**.**50** (3.25–13.00)	**18**.**00** (13.00–20.00)	**5**.**50** (3.25–14.00)
**LID (N** = **22)**	**25**.**50** (15.25–43.50)	**7**.**50** (3.75–10.75)	**13**.**50** (4.75–18.50)	**6**.**00** (4.00–11.00)
**NVD (N** = **24)**	**57**.**50** (45.75–70.50)	**19**.**00** (14.25–22.75)	**22**.**00** (16.25–28.75)	**16**.**00** (10.50–20.00)
**HVD (N** = **4)**	**32**.**00** (0.25–68.25)	**9**.**50** (0.25–16.00)	**12**.**00** (0.00–28.50)	**10**.**00** (0.00–20.75)
**Kruskal-Wallis H Test**	79.272	50.440	95.269	29.937
**P Level**	0.000*	0.000*	0.000*	0.000*

*p level at P < 0.05; Abbreviations: IQR, Interquartile Range; VHI: Voice Handicap Index, LML: Laryngeal Mass Lesions, LID: Laryngeal Inflammatory Disorders, NVD: Neurogenic Voice Disorders, HVD: Hyper-functional Voice Disorders.

Similarly, significant differences were found for the VHI-F domain [H (4) = 50.440, P < 0.001], with mean scores of 69.32 for non-dysphonic participants, 96.44 for LML patients, 99.39 for LID patients, 152.50 for NVD patients and 86.57 for HVD patients. Additionally, significant differences were identified for the VHI-P domain [H (4) = 95.269, P < 0.001], with mean scores of 55.61 for non-dysphonic participants, 130.19 for LML patients, 97.75 for LID patients, 149.38 for NVD patients and 85.50 for HVD patients. Finally, the VHI-E domain presented significant differences [H (4) = 29.937, P < 0.001], with mean scores of 75.32 for non-dysphonic participants, 90.95 for LML patients, 98.39 for LID patients, 139.69 for NVD patients and 89.00 for HVD patients (Table 3).

Additionally, a significant discriminatory ability was observed for the VHI between the VDP and the controls. Specifically, a very strong discriminatory ability was detected for the VHI-T scores (AUC 0.924, P < 0.001), VHI-F scores (AUC 0.849, P < 0.001), VHI-P scores (AUC 0.904, P < 0.001) and VHI-Ε scores (AUC 0.829, P < 0.001) (Table 4).

**Table 4:** Coordinates for VDP and Controls Curve for VHI Total Score and VHI Domains.

	AUC	SE	P level	95% CI
Total (VHI-T)	0.924	0.029	<0.001*	0.867–0.981
Functional (VHI-F)	0.849	0.038	<0.001*	0.775–0.923
Physical (VHI-P)	0.904	0.031	<0.001*	0.843–0.965
Emotional (VHI-E)	0.829	0.038	<0.001*	0.755–0.903

*p level at P < 0.05 Abbreviations: AUC, Area Under Curve; CI, Confidence Interval; SE, Standard Error; VHI, Voice Handicap Index.

A ROC analysis was conducted to determine the cut-off points of the VHI-T and its three domains (functional, physical and emotional). The threshold (cutoff point) was derived from the maximum “Sensitivity + Specificity” coordinates (1-Specificity, Sensitivity) (please see Krzanowski and Hand^47^). Another way of calculating the same value for the cutoff point similar is to find the maximum score obtained when subtracting “1-Specificity” from the“Sensitivity”, i.e., the cutoff point coordinates’ condition = the maximum {Sensitivity (i) – (1 – Specificity (i)} where i ϵ [n], [n] = {1, …, n}, for n ROC analysis points. The last can be justified as “Sensitivity” referring to true positives (i.e., voice disordered people are correctly identified as voice disordered) and “1-specificity” referring to false positives (i.e., people without disorders are incorrectly identified as voice disordered). Consequently, the best cutoff point should be the coordinates for which we have the maximum {Sensitivity (i) – (1 – Specificity (i)}, because that is the point at which the most people are identified correctly as voice disordered.

**Figure 1:**
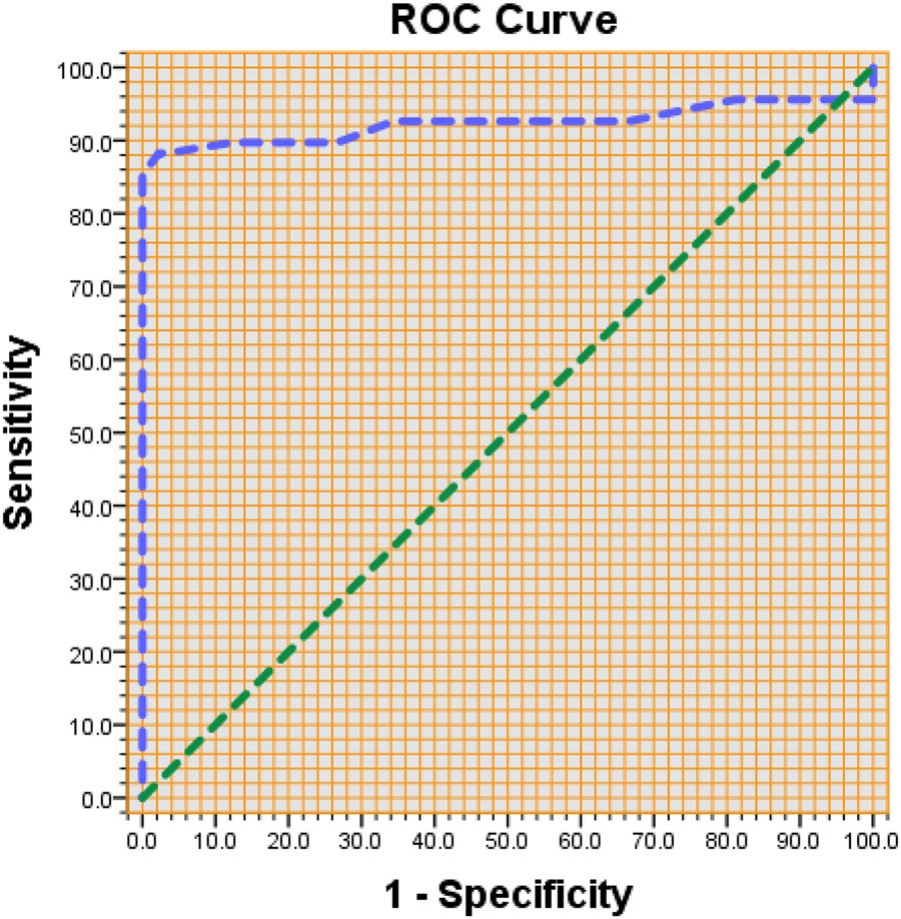
ROC Curve for the Voice Handicap Index – Total Score (VHI-T).

The cutoff point of the VHI-T score was found to be 19.50, with a sensitivity of 0.882 and a 1-specificity of 0.022 (Fig. 1). The VHI-F cutoff point was 7.50, with a sensitivity of 0.735 and a 1-specificity of 0.022. Additionally, the VHI-P cutoff point was 8.50, with a sensitivity of 0.868 and a 1-specificity of 0.000, and the VHI-E cutoff point was 8.50, with a sensitivity of 0.618 and a 1-specificity of 0.011. Specifically, with regard to the usage of the 3 domains’ cutoff values, these were considered in the past of lower importance compared to the total score. This could be partially correct when the domains are not equal. In contrast, when they are equal, which we observed for the first time in^45^, then this identifies with more certainty a potential voice problem, in addition to the fact that the self-perceived score is at least equal to the total cutoff score.

**Figure 2:**
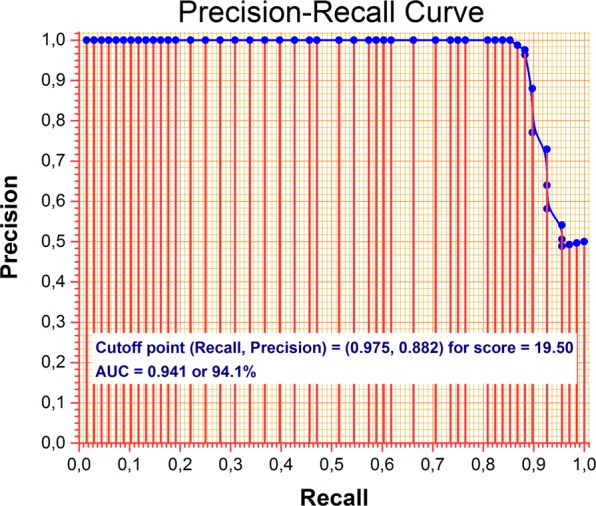
Precision-Recall Curve for the Voice Handicap Index – Total Score (VHI-T).

To validate the proposed total cut-off point (threshold) of 19.50, we decided to produce a precision-recall curve (Fig. 2). The main idea behind this dual analysis is that rare events (e.g., positive samples are very few in number compared to the negative ones), then a precision-recall analysis is appropriate^48^. This case does not apply to our sample but takes into consideration that the ROC curve remains unchanged relevant to “rare” positive samples, making it wise to conduct an additional validation of the already calculated ROC cutoff point. Consequently, after creating the precision-recall curve and finding the AUC of 94.1% with a cut-off point at 19.50, the threshold proposed in this study was validated for the detection of potential voice disorders in the Greek population.

## Discussion

The results of the present study (limitations apply, please see section 4.4) revealed that VDP exhibit significantly higher means and medians on all three domains and on the total score of the VHI compared to the control group. This finding indicates that dysphonic patients perceive their voice differently than non-symptomatic groups^15–17^. Consequently, the discriminatory ability of the VHI has been confirmed. Moreover, the following similarities and differences were found:

- For VDP, the VHI-T median and mean scores were estimated to be 35.50 and 37.82, respectively, which is similar to many results from studies of the cross-cultural adaptation of VHI to dysphonic populations^1,21,23–33^.
- The mean and the median of the Greek version of the VHI (mean = 37.00 and median = 32.00 - limitations apply, please see section 4.4)^25^ are almost the same as those of the original version of the VHI (mean = 33.69) and the Italian version of the VHI (mean = 38.38)^31^. The mean and median values of the VHI-F were 10.72 and 10.00, respectively, for the VDP subgroup. These scores were also in good agreement with those reported in other studies^1,21–33^ and were close to those on the original version of VHI (mean = 10.07)^1^ and the Greek VHI (mean = 10.00 and median = 9.00)^25^.
- Likewise, on the VHI-P, the mean and median scores were 16.98 and 18.00, respectively, which were nearly equal to those reported in previous studies^1,21–33^. Specifically, close similarity was found to the mean score reported by Jacobson *et al*. (mean score = 18.63)^1^, to the mean and median scores reported by Helidoni *et al*. (mean and median = 18.00)^25^ and to the mean score reported by Moradi *et al*. (mean = 19.98)^28^.
- Moreover, the VHI-E mean was 10.12, and the median was 8.00. These results are in agreement with the findings of previous research^1,21–33^. The Schindler *et al*. mean score on the VHI-E (equal to 10.63) was the closest to our study^31^, along with the Bonetti & Bonetti scores, which were a mean of 10.00 and a median of 8.00^23^.
- Additionally, for the LML, LID, NVD and HVD subjects, the mean and median scores on the VHI and of its three domains were approximately the same as those reported in previous studies of culturally adapted VHI^22–33^. This indicates that VHI clearly discriminates dysphonic from normal voice populations. Moreover, significant differences in VHI scores were observed between all VDP groups in comparison to those of the control group. This finding was observed in populations with voice disorders compared to non-dysphonic populations^1,21–36,41–43,46^, as well as in non-symptomatic smokers^18,44,45^ and other populations^15,16^. The aforesaid further demonstrates the discriminatory ability of the VHI.
- In this study, the cutoff point of the VHI-T score was 19.50 (in practice, this score should be at least equal to 19, as the minimum scale increase equals 1), which was within the cutoff value range of 12 to 20^30,40–46^ previously proposed^30,40–46^. Although there were only a few studies that determined the cutoff points for the VHI via ROC analysis for dysphonic populations^30,40–43,46^, the scores can be assumed to be accurate because they exhibit satisfactory AUCs, as in this study. The same cutoff point (VHI-T) was computed in a study of smokers. This VHI-T cut-off score was found via an ROC curve, with a high AUC^44,45^. Furthermore, a similar value to the Greek threshold was observed for the cut-off value of the VHI-T (equal to 20) relevant to the Swedish population^30^ and for a population with organic dysphonia after thyroidectomy, with a cutoff point of 18^43^.
- The Greek cutoff point (limitations apply, please see section 4.4) was equivalent to the Norwegian VHI-T cutoff point (19.00)^26^ and the threshold computed in Behlau *et al*.^40^ (19.00). Other studies determined lower cutoff points for the VHI-T compared to that in the present study. Moradi *et al*.^41^ identified a cut-off value of 14.5 using the Persian version of the VHI, which was not equal to value determined in this study. Likewise, a lower cutoff point for the VHI-T (15) was calculated by Van Gogh *et al*.^46^ using the Dutch VHI for patients with cancer in the glottic area and benign laryngeal lesions. Similarly, the German and the Polish version of the VHI exhibited different cutoff points (VHI-T = 12) compared to the Greek VHI cutoff point^29,42^. Most likely, the abovementioned lower scores were due to differences in the studied population^40^.

The previously mentioned studies did not consider the cutoff values of the VHI domains, only the total VHI score. The use of cutoff points for the individual VHI domains enhances the discriminatory ability and the diagnostic capability of this tool, which has also been reported before^41,42^. Karlsen *et al*.^26^ highlighted that the VHI-F score tends to accurately diagnostically discriminate between dysphonic and non-dysphonic individuals, which was also reported in the current study. Moreover, Helidoni *et al*.^25^ emphasized the fact that the VHI-P domain had a good discriminatory capability because VD patients are more familiar with vocal symptoms, and physical signs are more detectable through a self-perceived diagnosis.

The use of patient-centred evaluations, both objective and subjective, is of great importance, as concluded by other studies^4,25^. Specifically, the use of VHI cutoff points will help clinicians better understand the condition of people with vocal symptoms to better address their problems^25^ and to accurately monitor their health condition. To summarize the significant findings, we conclude the following (limitations apply, please see section 4.4), which are very important for accomplishing the globalization of the VHI and thus are related to the purposes of this study:

- The Greek VHI cutoff point (COP) was 19.50. Consequently, every score higher than this in the Greek population should immediately be reported to a specialist so that the potential patient can be examined.
- Various populations investigated in previous studies were also examined to determine commonalities with the Greek population. Most studies that met the following criteria exhibited similar cutoff points: the same type of sample (almost the same severity of disorders), nearly equal numbers of controls and patients, all the patients were in the same phase of therapy, and the study defined only a cutoff point and not a range.
- Taking into consideration the previous criteria, we found the following cutoff points: the Greek version reported by Tafiadis *et al*. for males and females (both COPs = 19.50)^44,45^, the Brazilian version (COP = 19)^40^, the Swedish version (COP = 20)^30^, the Norwegian version (COP = 19)^26^ and finally the American version for post-thyroidectomy patients (COP = 18)^43^. The mean cut-off point for these populations was found to be higher than 19 (i.e., 19.10) with a standard deviation of ≈0.7. In turn, this means that globally, when someone exhibits a higher score than 18.50, (in practice this score should be at least equal to 18 as the minimum scale increase is 1) then he/she should visit a specialist. This value is lower than the Greek cutoff point (in practice equal to 19), but it provides a more stringent preventive screening method.
- Τhis study demonstrated that the three domains of the VHI exhibit similar cut-off points for the Greek population and smokers^41,42^. This near equality between the subdomains (with cut-off points from 7.50 to 8.50) may be useful for regular monitoring in cases where there is a strong perception that a voice symptom is persistent. At the same time, the equality of the VHI thresholds may be beneficial for patients with voice problems who want to check their therapeutic progress, as has been suggested in the case of smoking cessation^41,42^.
- When people want to check themselves for potential voice disorders, the procedure is simple. They must fill out the VHI^1^. Then, they calculate their score. If their total score is above 18, then there is a strong possibility of a clinical voice problem. If so, they should seek the help of a professional. Especially for the Greek population, subdomains should also be checked. If their scores are above the cutoff points, then there is an even stronger possibility of a clinical voice disorder.
- Finally, a new factorial analysis could be performed, as in the research by Hongyan Li *et al*.^49^, who took into account various economic and cultural differences when they compared eastern and western populations and who designed a completely new method with new tools. However, these differences do not exist to a large extent between different western cultures. Consequently, there is no imminent need to redefine the VHI tool. Furthermore, our co-author Thomas Murry was the first to produce the VHI-10^50^ and he, along with our other co-author, Meropi E. Helidoni, introduced the formal Greek VHI edition^25^. Finally, members of the team published in 2018 the formal Greek VHI-10 edition^51^. Consequently, based on a deep knowledge of the VHI, we declare that there is no need to re-evaluate the VHI tool. Nevertheless, factorial analysis could be useful in the future to determine how much our cultures have changed over time being due to economic issues and population migration. The last two facts could even constitute new balances and behaviours inside newly emerged societies.

### Conclusions

The aim of this study was to estimate the VHI cutoff points (for all domains) for populations with voice disorders (VD) in Greece (limitations apply, please see section 4.4). The VHI can significantly distinguish VD and non-VD populations based on their perception of their voice status. The Greek VHI can be used as a monitoring tool for clinicians. Furthermore, it can be used as a screening procedure for voice professionals. The proper use of the VHI cut-off points can assist a voice clinician in better predicting the course of a patient’s voice disorder. Finally, these cutoff points, in combination with a voice assessment, can better characterize an individual’s voice disorder. In turn, this can lead to a more customized treatment.

## Materials and Methods

### Participants

This study was approved by the Ethical Committee of the Medical School-University of Crete. One hundred eighty participants (90 non-dysphonic and 90 dysphonic patients) were enrolled in this study. Particularly, the sample was consisted of 42 non-dysphonic men, 48 non-dysphonic women, 40 dysphonic men and 50 dysphonic women. The mean age of the total sample was 43.32 yrs. (SD = 14.60) with a male mean age of 43.86 yrs. (SD = 15.20) and a female mean age of 42.93 yrs. (SD = 14.21). The mean age of the control group was 38.04 yrs. (SD = 12.00) and it ranged from 22 to 74 yrs., with a male mean age of 37.25 yrs. (SD = 11.12) and a female mean age of 38.86 yrs. (SD = 13.83). The mean age of the patients with voice disorders (VDP) was 49.19 yrs. (SD = 15.01 years), ranging from 26 to 74 years, with a male mean age of 66.67 yrs. (SD = 5.05) and a female mean age of 59.42 yrs. (SD = 13.32).

All subjects with voice disorders were classified by ENT doctors and speech language pathologists (SLPs). The patients’ subgroup was determined via video laryngeal endoscopy and stroboscopy. All VDP had not undergone a previous laryngeal surgery nor did they have other recent surgeries during recruitment. Sixty-seven (67) patients attended the ENT clinic of the University Hospital of Crete, while the remaining twenty-three (23) attended a private medical office in Athens-Greece and an SLP office in Ioannina-Greece. The geographic distribution of normative subjects (90 non-dysphonic) was the same compared to dysphonic subjects.

All VDP were split into four diagnosed subgroups. The first subgroup was the “Laryngeal Mass Lesions (LML)” group (40 patients), with twenty-two patients (22) diagnosed with vocal nodules, fourteen (14) with vocal polyps, two (2) with vocal fold cysts and two (2) with leucoplakia. The second subgroup was the “Laryngeal Inflammatory Disorders (LID)” group (22 patients), with ten (10) patients diagnosed with Reinke’s oedema, two (2) with vocal fold haemorrhage and ten (10) with chronic laryngitis. The third subgroup was the “Neurogenic Voice Disorders (NVD)” group (24 patients), with ten (10) patients diagnosed with vocal fold paralysis, two (2) with vocal fold paresis, one (1) with superior laryngeal nerve paresis, one (1) with spasmodic dysphonia and ten (10) with voice changes due to hypokinetic dysarthria (Parkinson’s disease). Finally, the “Hyper-functional Voice Disordered (HVD)” group consisted of 4 patients with muscle tension dysphonia.

The control group consisted of subjects who accompanied the patients, those attending the ENT department for reasons other than voice disorders, clinical staff members and subjects from the School of Health and Welfare Professions at the TEI of Epirus.

Non-dysphonic subjects who were not included in the study presented any upper or lower respiratory system disorder or any laryngeal/vocal complaints in the last two weeks or had symptoms of gastroesophageal reflux (GERD) or laryngopharyngeal reflux (LRP) disease. In addition, excluded subjects were those who had attended voice therapy and/or presented voice disorders in the past, who had a history of alcohol and/or drug abuse, who lived or worked in environments that had factors influencing the voice (smoke, dust, exposure to chemicals, external noise, and/or allergens, etc.).

It must also be clarified that the number of participants was defined based only on the strict criteria selection and not on statistical estimations of the sample size. Consequently, many participants were found, but a large number was excluded.

The population sample was partially representative of the general population of Greece (limitations apply, please see section 4.4), and the distributions of pathology, gender and age were also appropriate. Particularly while the sample size seems small, many people were excluded based on the strict criteria mentioned in this section, which balances the drawback of the small total number. Moreover, (1) because the sample originated from different regions of Greece, it is partially representative (locations were only from Athens, Ioannina, Crete); (2) the sample was selected with strict exclusion criteria, which indicates additional sample representativeness; and (3) as the sample’s collection was conducted by different independent professional researchers who collected this sample of each region, the sampling procedure could be defined as adequate with limitations (please see section 4.4).

### Data collection

The Hellenic VHI index^25^ and the translation of the Greek Voice Evaluation Template (VET)^52^ were filled in by all participants. The VET is a consensus template about the voice that the American Speech Hearing Association (ASHA) developed that can be used in daily clinical practice. All subjects received information before enrolment about the study’s purposes and the confidentiality of the obtained data. They were also asked to sign a consent form.

### Statistical analysis

The distribution of variables was tested with the Kolmogorov-Smirnov and Shapiro-Wilk tests. All skewed variables (VHI scores) are expressed as the medians and the interquartile range, and all normally distributed variables are expressed as the means and standard deviations (SD). A Mann-Whitney U test was used for the comparison of the two study groups (dysphonic and non-dysphonic participants). Furthermore, the Kruskal-Wallis test was used to compare the five subgroups (non-dysphonic participants, LML patients, LID patients, NVD patients and HVD patients). ROC and precision-recall analyses were conducted to estimate the best cut-off values for the VHI-Total (T) and its three domains. All reported p-values were two-tailed, and the statistical significance was set at the value of p < 0.05. The analysis was performed with SPSS statistical software (version 19.0, Armonk, NY, USA).

### Limitations and future solutions

The sample collection was conducted only in three different regions of Greece (Athens, Ioannina, Crete) so this constitutes a limitation of this study. Future works should include more regions in order the sample to be characterized as Panhellenic and not as partially representative like in our study.

Moreover, most of the dysphonic samples (67) were collected from the University Hospital of Crete while the remaining twenty-three (23) attended a private medical office in Athens-Greece and an SLP office in Ioannina-Greece. At first sight the sample seems strongly representative of the Crete’s population and not generally all of Greece, but this is not the case as we conducted various random checks to people all around Greece compared to the sample of this candidate paper. Unfortunately the gathered sample from each area was too small, so this does not constitute a strict validity in order to give further details in the manuscript. A future solution to this limitation should include a vast and well-balanced sample collection from many regions of Greece. The sample should be gathered in such a way that should depend on Eurostat data (the number of participants from every region should correspond to the real percentage of the Greek living population in these areas). Then this future study should be definitely treated as an epidemiological study.

### Ethical approval

All procedures performed in studies involving human participants were conducted in accordance with the ethical standards of the institutional and/or the national research committee and with the 1964 Helsinki declaration and its later amendments or comparable ethical standards.

### Informed consent

Informed consent was obtained from all individual participants included in the study. This study was approved by the Ethical Committee of the Medical School-University of Crete.

## Data Availability

All the included data are fully available without restriction.

## References

[1] Jacobson BH (1997). The Voice Handicap Index (VHI). Am J Speech Lang Pathol..

[2] Olthoff A (2003). Assessment of Irregular Voices After Total and Laser Surgical Partial Laryngectomy. Arch Otolaryngol Head Neck Surg..

[3] Wuyts FL (2000). The Dysphonia Severity Index. J Speech Lang Hear Res..

[4] Dejonckere PH (2001). A basic protocol for functional assessment of voice pathology, especially for investigating the efficacy of (phonosurgical) treatments and evaluating new assessment techniques. Eur Arch Otorhinolaryngol..

[5] DeJonckere PH (2003). Implementation of the European Laryngological Society (ELS) basic protocol for assessing voice treatment effect. Rev Laryngol Otol Rhinol..

[6] Beser M (2009). Detection of laryngeal tumors and tumoral extension by multislice computed tomography-virtual laryngoscopy (MSCT-VL). Eur Arch Otorhinolaryngol..

[7] Klinge K, Guntinas-Lichius O, Axtmann K, Mueller AH (2015). Synchronous video laryngoscopy and sonography of the larynx in children. Eur Arch Otorhinolaryngol..

[8] Bless DM, Glaze LE, Lowery DB, Campos G, Peppard RC (1993). Stroboscopic, acoustic, aerodynamic, and perceptual analysis of voice production in normal speaking adults. NCVS Status and Progress Report.

[9] Arias MR, Ramón JL, Campos M, Cervantes JJ (2000). Acoustic analysis of the voice in phonatory fistuloplasty after total laryngectomy. Otolaryngol Head Neck Surg..

[10] Campos RJ, Maciel CT, Cesca MG, Leite IC (2011). Voice analysis after cancer treatment with organ preservation. Head Neck Oncol..

[11] Fernández RL (1999). Acoustic analysis of the normal voice in nonsmoking adults. Acta Otorrinolaringol Esp..

[12] Hummel C, Scharf M, Schuetzenberger A, Graessel E, Rosanowski F (2010). Objective Voice Parameters and Self-Perceived Handicap in Dysphonia. Folia Phoniatr Logop..

[13] Kotby, M. N. *et al*. Aerodynamic analysis of voice disorders. *Arch Otolaryngol Head Neck Surg Proc 14th World Congr Otorhinolaryngol Head Neck Surg*. **3** (1990).

[14] Lovato A (2016). Multi-Dimensional Voice Program (MDVP) vs Praat for Assessing Euphonic Subjects: A Preliminary Study on the Gender-discriminating Power of Acoustic Analysis Software. J Voice..

[15] Tafiadis D (2017). Acoustic and Perceived Measurements Certifying Tango as Voice Treatment Method. J Voice..

[16] Tafiadis D (2017). Comparison of Voice Handicap Index Scores Between Female Students of Speech Therapy and Other Health Professions. J Voice..

[17] Tafiadis D, Tatsis G, Ziavra N, Toki EI (2017). Voice Data on Female Smokers: Coherence between the Voice Handicap Index and Acoustic Voice Parameters. AIMS Med Sci..

[18] Tafiadis D, Toki EI, Miller KJ, Ziavra N (2017). Effects of Early Smoking Habits on Young Adult Female Voices in Greece. J Voice..

[19] Vilaseca I, Blanch JL, Bernal-Sprekelsen M, Moragas M (2004). CO2 laser surgery: A larynx preservation alternative for selected hypopharyngeal carcinomas. Head Neck..

[20] Vilaseca I (2008). Voice quality after CO2 Laser cordectomy—what can we really expect?. Head Neck..

[21] Amir O (2006). Applying the Voice Handicap Index (VHI) to Dysphonic and Nondysphonic Hebrew Speakers. J Voice..

[22] Behlau M, Alves Dos Santos Lde M, Oliveira G (2011). Cross-Cultural Adaptation and Validation of the Voice Handicap Index Into Brazilian Portuguese. J Voice..

[23] Bonetti A, Bonetti L (2013). Cross-cultural adaptation and validation of the Voice Handicap Index into Croatian. J Voice..

[24] Hakkesteegt MM, Wieringa MH, Gerritsma EJ, Feenstra L (2006). Reproducibility of the Dutch Version of the Voice Handicap Index. Folia Phoniatr Logop..

[25] Helidoni ME (2010). Cross-Cultural Adaptation and Validation of the Voice Handicap Index Into Greek. J Voice..

[26] Karlsen T, Grieg ARH, Heimdal JH, Aarstad HJ (2012). Cross-Cultural Adaption and Translation of the Voice Handicap Index into Norwegian. Folia Phoniatr Logop..

[27] Malki KH, Mesallam TA, Farahat M, Bukhari M, Murry T (2010). Validation and cultural modification of Arabic voice handicap index. Eur Arch Otorhinolaryngol..

[28] Moradi N (2013). Cross-Cultural Equivalence and Evaluation of Psychometric Properties of Voice Handicap Index Into Persian. J Voice..

[29] Niebudek-Bogusz E, Kuzańska A, Woznicka E, Sliwinska-Kowalska M (2011). Assessment of the Voice Handicap Index as a Screening Tool in Dysphonic Patients. Folia Phoniatrica et Logopaedica..

[30] Ohlsson AC, Dotevall H (2009). Voice handicap index in Swedish. Logop Phoniat Vocol..

[31] Schindler A (2010). Cross-cultural Adaptation and Validation of the Voice Handicap Index Into Italian. J Voice..

[32] Taguchi A, Mise K, Nishikubo K, Hyodo M, Shiromoto O (2012). Japanese Version of Voice Handicap Index for Subjective Evaluation of Voice Disorder. J Voice..

[33] Trinite B, Sokolovs J (2014). Adaptation and validation of the Voice Handicap Index in Latvian. J Voice..

[34] Stuut M, Robin EA, Tjon Pian G, Dikkers FG (2013). Change of Voice Handicap Index after treatment of benign laryngeal disorders. Eur Arch Otorhinolaryngol..

[35] Batalla FN (2007). Voice quality after endoscopic laser surgery and radiotherapy for early glottic cancer: objective measurements emphasizing the Voice Handicap Index. Eur Arch Otorhinolaryngol..

[36] Hsiung MW, Pai L, Wang HW (2002). Correlation between voice handicap index and voice laboratory measurements in dysphonic patients. Eur Arch Otorhinolaryngol..

[37] Toki Eugenia I., Plachouras Konstantinos, Tatsis Georgios, Chronopoulos Spyridon K., Tafiadis Dionysios, Ziavra Nausica, Siafaka Vassiliki (2018). The Design of a Mobile System for Voice e-Assessment and Vocal Hygiene e-Training. Advances in Intelligent Systems and Computing.

[38] Toki Eugenia I., Tafiadis Dionysios, Rizos Konstantinos, Primikiri Marina, Tatsis Georgios, Ziavra Nausica, Siafaka Vassiliki (2018). A Preliminary Study on a Mobile System for Voice Assessment and Vocal Hygiene Training: The Case of Teachers. Advances in Intelligent Systems and Computing.

[39] Toki Eugenia I., Siafaka Vassiliki, Moutselakis Dimitrios, Ampatziadis Prodromos, Tafiadis Dionysios, Tatsis Georgios, Ziavra Nausica (2018). A Preliminary Study on a Mobile System for Voice Assessment and Vocal Hygiene Training in Military Personnel. Advances in Intelligent Systems and Computing.

[40] Behlau M (2016). Efficiency and Cutoff Values of Self-Assessment Instruments on the Impact of a Voice Problem. J Voice..

[41] Moradi N, Pourshahbaz A, Soltani M, Javadipour S (2013). Cutoff Point at Voice Handicap Index Used to Screen Voice Disorders Among Persian Speakers. J Voice..

[42] Gräßel E, Hoppe U, Rosanowski F (2007). Graduierung des Voice-Handicap-Index. Hno.

[43] Solomon NP (2013). Utility of the Voice Handicap Index as an Indicator of Postthyroidectomy Voice Dysfunction. J Voice..

[44] Tafiadis D (2018). Voice Handicap Index and Interpretation of the Cutoff Points Using Receiver Operating Characteristic Curve as Screening for Young Adult Female Smokers. J Voice..

[45] Tafiadis D (2017). Using Receiver Operating Characteristic Curve to Define the Cutoff Points of Voice Handicap Index Applied to Young Adult Male Smokers. J Voice..

[46] Gogh CDLV (2007). Voice in early glottic cancer compared to benign voice pathology. Eur Arch Otorhinolaryngol..

[47] Krzanowski, W. J. & Hand, D. J. ROC *curves for continuous data*. (Chapman and Hall/CRC 2009).

[48] Davis, J. & Goadrich, M. The relationship between Precision-Recall and ROC curves. *In Proceedings of the 23rd international conference on Machine learning*. AMC. 233–240, 10.1145/1143844.1143874 (2006).

[49] Li H, Huang Z, Hu R, Zhang L, Xu W (2012). Study on the simplified Chinese version of the voice handicap index. J Voice.

[50] Rosen CA, Lee AS, Osborne J, Zullo T, Murry T (2004). Development and validation of the voice handicap index**-**10. The Laryngoscope..

[51] Tafiadis, D. *et al*. Cross-cultural Adaptation and Validation of the Greek Voice Handicap Index-10 (GVHI-10) With Additional Receiver Operating Characteristic Analysis. *J Voice*. In Press, 10.1016/j.jvoice.2018.09.009 (2018). 10.1016/j.jvoice.2018.09.009 30301578

[52] American Speech Hearing Association. Voice Evaluation Template, https://www.asha.org/uploadedFiles/AATVoiceEvaluation.pdf (2015).

